# Synthesis, characterization, and anticancer potency of sulfadiazine salicylaldehyde-based Schiff bases

**DOI:** 10.1038/s41598-026-51752-z

**Published:** 2026-05-13

**Authors:** Esraa Nasr, Ahmed H. Moustafa, Ashraf S. A. El-Sayed, Mohamed Ali

**Affiliations:** 1 Biochemistry Department, Faculty of Science, Zagazig University, Zagazig, 44519 Egypt; 2 Chemistry Department, Faculty of Science, Zagazig University, Zagazig, 44519 Egypt; 3 Enzymology and Fungal Biotechnology Lab, Botany and Microbiology Department, Faculty of Science, Zagazig University, Zagazig, 44519 Egypt

**Keywords:** Schiff bases, Anticancer activity, Apoptosis, Molecular docking, Biochemistry, Cancer, Chemical biology, Chemistry, Drug discovery

## Abstract

Cancer is a major worldwide health issue, with rising prevalence and limitations to traditional chemotherapy demanding the development of innovative, selective anticancer drugs. Schiff bases have emerged as promising candidates due to their diverse biological and anticancer activities. The present study aims to emphasize the chemical identity and anticancer activity of (E)-4-(2-hydroxybenzylideneamino)-N-(pyrimidin-2-yl) benzene-sulfonamide; SB1 and 4-(5-bromo-2-hydroxybenzylideneamino)-N-(pyrimidin-2-yl)-benzenesulfonamide); SB2. The structural identity was determined by FTIR 460 PLUS and ^1^H NMR spectra. The cytotoxicity of SB1 and SB2 were evaluated using MTT against various human cancer cells including HCT116, MCF-7, A549, HepG2, T24 compared to WI-38 cells. Additional further studies on MCF-7 and HCT116 cells, including Annexin V-FITC apoptosis detection, ROS generation, DNA fragmentation, and Topoisomerase I and II inhibition, were carried out to clarify their modes of action. Molecular docking studies were performed to predict how the produced compounds attached to certain target proteins linked to cancer, shedding light on their putative molecular mechanisms of action. The results showed that the IC_50_ of MCF-7, treated with SB1, exhibited the strongest antiproliferative effect (66.27 ± 1.14 µg/mL), followed by HCT116 (90.01 ± 0.12 µg/mL), A549 cells (91.5 ± 2.06 µg/mL), T24 (192.28 ± 1.12 µg/mL), and HepG-2 cells (240.08 ± 3.83 µg/mL). Furthermore, SB2 had the highest antiproliferative activity of MCF-7 (59.61 ± 1.59 µg/mL), followed by HCT116 (48.65 ± 2.12 µg/mL), A549 cells (68.56 ± 1.6 µg/mL), T24 (216.13 ± 0.7 µg/mL), and HepG-2 cells (120.53 ± 2.62 µg/mL). Moreover, SB1 and SB2 cause oxidative stress and apoptosis in cancer cells compared to the control. The molecular docking results showed that the Schiff bases have strong in vitro antineoplastic action and have high binding affinities for key proteins involved in cancer progression. These findings highlight the value of these two compounds of Schiff base derivatives as lead molecules in the development of tailored anticancer drugs.

**Supplementary Information:**

The online version contains supplementary material available at 10.1038/s41598-026-51752-z.

## Introduction

Cancer is still one of the leading causes of death worldwide. Following cardiovascular disease, it was mentioned that the global cancer burden is expected to be 28.4 million cases, with a 47% increase from 2020 to 2040^1–3^. Cancer is defined as a multi-step progressive disease in which some cells begin to proliferate in an uncontrolled manner as a result of a major alteration in the cellular genetic makeup. Regulation of cellular genes, cellular proliferation, and cell cycles get altered, transforming the normal cell into a malignant one^4^.These cancer cells bypass cell cycle checkpoints, avoid apoptosis, create vast populations, grow in size, and invade neighboring tissues^5^.

Cancer can be treated and controlled efficiently if found in the early stages, but in undiagnosed situations, it may become lethal and the disease advances to an end stage^6^. In cancer treatment, several principles-based therapies such as radiotherapy, chemotherapy, and surgical procedures are now used. Chemotherapy is one of the most widely used cancer treatments^7^. Today, the various chemotherapeutic drugs in cancer chemotherapy are used in clinic applications^8^. The side effects of these drugs vary depending on the type and dose of drugs administered, as well as the period of time they are taken. Despite substantial breakthroughs in chemotherapy, the unfavorable side effects and disadvantages have encouraged us to investigate novel chemotherapeutic agents. As a result, much research is required to discover effective anticancer drugs that can target the biochemical markers of cancer cells. Cancer cells can avoid death by deregulating their apoptotic machinery, allowing them to become immortal. Thus, the efficacy of cancer chemotherapy is dependent on the specific induction of apoptosis in malignant cells^9^.

Several researchers have demonstrated the biological significance of many structural derivatives of heterocyclic Schiff bases, which are important chemical compounds that have a variety of applications. The Schiff bases are characterized by an imine or azomethine (–C=N–) group. They are generated via the condensation process between carbonyl molecules (aldehydes or ketones) and compounds with an amine group^10,11^.

Furthermore, Schiff bases derived from various heterocyclic scaffolds have been reported to exhibit a wide spectrum of pharmacologic activities, including antimicrobial^12^, anthelmintic, analgesic^13^, anti-inflammatory, allergen-reducing activity^14^, antipyretic^15^, diuretic, hypoglycemic^16^, and antitumor activities^17,18^. Furthermore, these compounds have demonstrated a notable antioxidant potential, which has been attributed to their radical scavenging capabilities^19,20^

Most bioactive compounds contain heterocycles, which play an important role in their bioactivity. Aromatic primary amines may also contain other donor functional groups such as –Br, –OH, –CH3, and so on, which aid in the enhancement and regulation of biological activities^21,22^. The presence of the imine (C=N) group contributes to the diverse chemical reactivity and biological activity of Schiff base compounds^10^. Because of the biological activity of Schiff bases, stimulating the apoptotic process in tumor cells is a promising goal for developing a novel drug against a variety of cancer cells during both in vitro and in vivo research.

In the present study, two Schiff bases, SB1 and SB2, were synthesized and evaluated for their antiproliferative activity against a panel of human cancer cell lines. To elucidate their underlying mechanisms of action, a series of biological assays were performed, including apoptosis analysis, reactive oxygen species (ROS) generation, DNA fragmentation, and topoisomerase inhibition studies. In addition, molecular docking was conducted to investigate the binding interactions of SB1 and SB2 with carbonic anhydrase XII and the ATPase domain of human DNA topoisomerase II, thereby assessing their potential as anticancer agents.

## Material and methods

### Chemical and reagents

The study was carried out using high-quality materials. Sigma-Aldrich provided all of the chemicals (sulfadiazine, salicylaldehyde, 5-bromo-2-hydroxy benzaldehyde, glacial acetic acid) (Taufkirchen, Germany). El-Nasr Pharmaceutical Chemicals Company supplied all solvents (ethanol 99.8%, DMSO 99%) (analytical reagent grade, Egypt). Melting points were measured using an uncorrected Cole-Parmer digital Electrothermal IA 9100 Series (Beacon Road, Stone, Staffordshire, ST15 OSA, UK). The FTIR 460 PLUS was used to produce IR spectra (KBr discs). The ^1^H NMR and the ^13^C NMR spectra were generated using a Bruker 400 MHz NMR Spectrometer with DMSO-d6 as the solvent.

### Synthesis of Schiff base SB1 and SB2

(E)-4-(2-hydroxybenzylideneamino)-*N*-(pyrimidin-2-yl) benzene-sulfonamide (HBAPBS) Schiff base (SB1) was synthesized as previously described by^23,24^ . Briefly, 15 mL of an ethanolic solution of salicylaldehyde (0.005 mol) was mixed with 30 mL ethanolic solution of 4-amino-*N*-(pyrimidin-2-yl) benzene sulfonamide (0.005 mol). The combination was refluxed in a water bath for 3 h. Following room temperature cooling, the precipitate was filtered, collected, and thrice washed with 20 ml of ethanol. It was dried in the presence of air, and recrystallization with ethanol, yield 96.6%***.*** The yellow crystal formed has a melting point ranging from 240 to 242 °C.

**Scheme 1:**
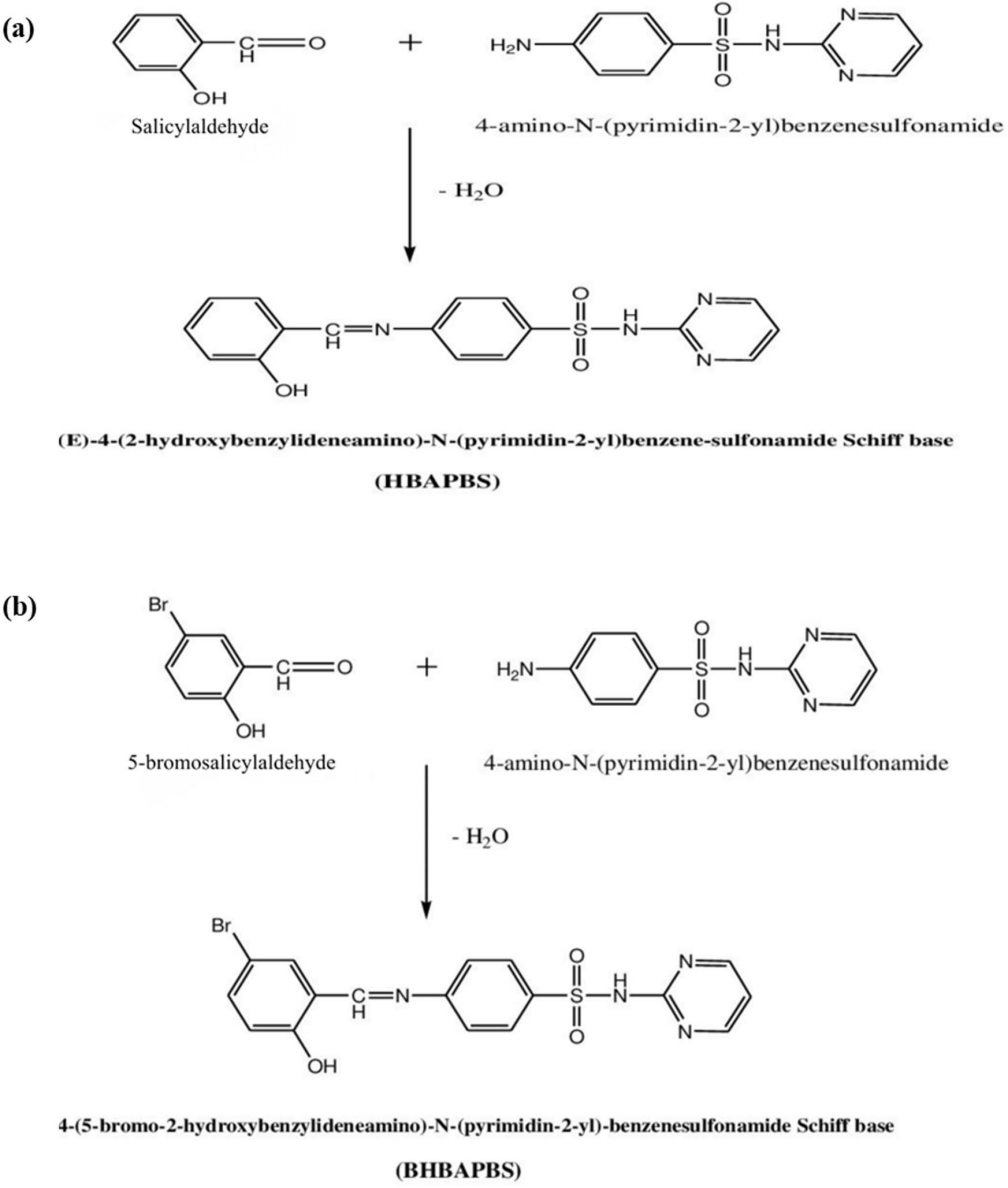
(**a**) Schematic representation of the synthesis of the Schiff base (E)-4-(2-hydroxybenzylideneamino)-N-(pyrimidin-2-yl) benzenesulfonamide (HBAPBS). (**b**) Schematic representation of the synthesis of the Schiff base 4-(5-bromo-2-hydroxybenzylideneamino)-N-(pyrimidin-2-yl) benzenesulfonamide (BHBAPBS).

Additionally, 4-(5-bromo-2-hydroxybenzylideneamino)-N-(pyrimidin-2-yl) benzenesulfonamide (BHBAPBS) Schiff base (SB2) was synthesized using a procedure similar to that described for SB1. Briefly, 15 mL of an ethanolic solution of 5-bromo-2-hydroxybenzaldehyde (0.005 mol) was added to 30 mL of an ethanolic solution of 4-amino-N-(pyrimidin-2-yl) benzenesulfonamide (0.005 mol). The reaction mixture was refluxed in a water bath for 3 h. After cooling to room temperature, the resulting precipitate was filtered, collected, and washed three times with 20 mL portions of ethanol. The product was air-dried and recrystallized from ethanol, giving a 97% yield. The obtained orange crystals had a melting point of 290–292 °C. The synthesis procedure is illustrated in Scheme 1.

### Characterization of the synthesized Schiff base compounds

The synthesized compounds SB1 and SB2 were characterized using The FTIR 460 PLUS was used to produce IR spectra (KBr discs). The ^1^H NMR and ^13^C NMR spectra were generated using a Bruker 400 MHz NMR Spectrometer with DMSO-d_6_ as the solvent at Applied Nucleic Acids Research Center, Faculty of Science, Zagazig University.

^1^H NMR (DMSO-d₆, 400 MHz) spectrum of SB1 and SB2.

***SB₁:*** IR (KBr): 3164, 1619 and 1339 cm^-1^ for (NH, C==N and asym. SO_2_). ^1^H NMR (DMSO-d_6_): δ = 6.1 (s, 1H, NH, exchange with D_2_O), 6.5 (s, 1H, Ar–H), 7.11 (m, 2H, Ar–H), 7.3–7.6 (m, 5H, Ar–H), 8.1 (s, 1H, Ar–H), 8.5 (d, 2H, Ar–H), 8.9 (s, 1H, CH=N) and 12.5 (s, 1H, OH, exchange with D_2_O). Elemental analyses of the HBAPBS (C_17_H_14_N_4_O_3_S; M.Wt. 354.38); C, 57.59; H, 3.89; N, 15.86. Found (%): C, 57.56; H, 3.90; N, 15.89.

***SB₂:*** IR (KBr): 3432, 3123, 1619 and 1336 cm^-1^ for (OH, NH, C==N and asym. SO_2_). ^1^H NMR (DMSO-d_6_): δ = 6.1 (s, 1H, NH, exchange with D_2_O), 6.51 (s, 1H, Ar–H), 7.0 (m, 2H, Ar–H), 7.29–7.61 (m, 4H, Ar–H), 8.1 (s, 1H, Ar–H), 8.56 (d, 2H, Ar–H), 9.0 (s, 1H, CH=N) and 12.49 (s, 1H, OH, exchange with D_2_O). Elemental analyses of the BHMAPBS (C_17_H_13_BrN_4_O_3_S; M.Wt. 433.28); C, 47.21; H, 3.08, N, 12.89. Found (%): C, 47.33; H, 3.05, N, 12.85.

The synthesized compounds SB1 and SB2 were characterized by ^13^C NMR spectroscopy in DMSO-d₆ at 100.6 MHz. The ^13^C NMR spectrum of SB1 exhibited δ = 112.6, 115.9, 117.1, 117.6, 119.8, 122.1, 122.7, 129.5, 129.7, 130.2, 134.4, 136.8, 138.4,157.7, 158.7, 165.8, and 172.4 (Ar–C and C=N). Similarly, SB2 displayed 13C NMR (DMSO-d6): δ = 112.6, 115.9, 116.2, 119.6, 120.3, 121.8, 129.5, 130.2,130.9, 134.3, 157.7, 158.8, 160.3, 163.8 and 172.4 (Ar–C and C=N). The observed spectral data are consistent with the proposed structures of SB1 and SB2.

### Cytotoxic effect of the synthesized Schiff Base against human cancer cell lines

The cytotoxic effect of SB1 and SB2 was evaluated against a panel of human cancer cell lines, including colon carcinoma (HCT116), breast carcinoma (MCF-7), Hepatocellular carcinoma (HepG2), lung carcinoma (A549), and urinary bladder carcinoma (T24), alongside the normal lung epithelial cell line WI-38. All cell lines were acquired from the American Type Culture Collection (ATCC, Manassas, VA, USA). With 10% fetal bovine serum (FBS, BioSolutions International, Melbourne, Australia), 1% penicillin–streptomycin mixture (Invitrogen, Grand Island, NY, USA), and 1% L-glutamine (Sigma-Aldrich, Inc.), the cells were cultured in fresh Dulbecco’s Modified Eagle’s Medium (DMEM, Sigma-Aldrich, Inc., St. Louis, MO, USA) at 37 °C in a humidified 5% CO2 atmosphere. The antiproliferative activity of SB1 and SB2 was evaluated utilizing the MTT Assay, as explained by^25^. Briefly, A 96-well tissue culture plate was seeded with 1 × 10^4^ cells/well at 37 °C for 24 h to form a full monolayer. The tested compounds were added at different concentrations and incubated for 48 h, then 20 µl of MTT solution (5 mg/ml in PBS, obtained from BIO BASIC CANADA INC) was added to each well. The plate was incubated at 37 °C with 5% CO₂ for 4 h, allowing live cells to metabolize MTT. Following incubation, the media was carefully removed. MTT metabolism produced formazan, which was then resuspended in 200 µl of DMSO. After that, the plate was shaken at 150 rpm for five minutes to fully mix the formazan with the solvent. Ultimately, the IC_50_ value was calculated by measuring the optical density (OD) at 570 nm^26^.

### Flow cytometric analysis of apoptosis using annexin V-FITC/PI

The cellular apoptotic processes due to the treatment of HCT116 and MCF-7 cells with the synthesized Schiff bases was examined by Annexin V-FITC Apoptosis Kit (Catalog #: ab139418) as described by^27^, that based on externalization of the plasma membrane phosphatidylserine (PS) from the inner face to the cell surface, that subsequently form Annexin V-PS conjugates. Following a two-day incubation period under conventional cultural conditions, the cells were treated with the synthesized Schiff base at IC_50_ concentrations. Following cell harvesting and PBS washing, 500 µl of 1X binding buffer was used to resuspend the cells. The cell solution was then supplemented with 5 µl of Annexin V-FITC and 5 µl of propidium iodide. The mixture was left in the dark for 15 min at room temperature. With a FITC signal detector, the conjugates of Annexin V-PS were detected at Ex λ488 nm and Em λ530 nm. Following the identification of the various cell types, cells that were FITC negative and PI negative were classified as viable, FITC positive and PI negative as early apoptotic, FITC positive and PI positive as necrotic or late apoptotic, and FITC negative and PI positive as necrotic. BD FACSCanto II (BD Biosciences, BD FACS Diva 6.2.1 software) was used to analyze the data.

### Determination of apoptotic markers

The levels of pro-apoptotic markers (Cytochrome C, BAX, Caspase-9, caspase-3, caspase-7) and the anti-apoptotic marker (Bcl-2) were measured using ELISA kits from Invitrogen (Catalog No. KHO1051, EEL030, BMS2025TEN, KHO1091, EH71RB, and BMS244-3 respectively) according to the manufacturer’s instructions. The assay is based on antigen–antibody specificity, forming a sandwich complex detected via a colorimetric reaction. Optical density was measured at 450 nm using a BioTek ELx800 microplate reader. Protein concentrations (ng/mL) were determined from standard calibration curves. All samples were analyzed in Triplicate and expressed as mean ± SD.

### Reactive oxygen species assay

The ROS generation in HCT116 and MCF-7 cells were measured using a flow cytometer and a fluorometric assay kit (catalog No: E-BC-K138-F). Following a 6-h treatment with the synthesized Schiff base compounds, the cells were collected, rinsed with PBS, resuspended in PBS containing 20 μM DCFDA, and incubated in the dark for 30 min. The sample was further mixed with 300 µl of PBS, and the BD FACSCanto II flow cytometer (BD FACS Diva 6.2.1 software; BD Biosciences) was used to record the fluorescent intensity^28^.

### Quantitation of fragmented DNA using diphenylamine (DPA)

DNA fragmentation following SB1 and SB2 treatment on HCT116 and MCF-7 cells was carried out according to the method described by^29^. To put it briefly, the HCT116 and MCF-7 cells were exposed to SB1 and SB2 for 48 h after being plated at a concentration of roughly 1 × 10^6^, centrifuged at 2000 × g for 10 min at 4 °C to collect them. The cell pellets were lysed in 0.5 ml of lysis buffer that included 10 mM tris–HCl (pH 8), 1 mM EDTA, 0.2% Triton X-100 and centrifuged at 10,000 rpm for 20 min at 4 °C. 0.5 ml of lysis buffer were used to resuspend the pellets. After adding 0.5 ml of 25% trichloroacetic acid (TCA) to the pellets (P) and supernatants (S), they were incubated for 24 h at 4 °C. After centrifuging the samples for 20 min at (10,000 rpm) at 4 °C, the pellets were suspended in 80 μl of 5% TCA and incubated for 20 min at 83 °C. Each sample was then incubated for 24 h at room temperature after receiving 160 μl of DPA solution (150 mg DPA in 10 ml glacial acetic acid, 150 μl sulfuric acid, and 50 μl acetaldehyde (16 mg/ml)) (Burton, 1956). The proportion of fragmented DNA was calculated from absorbance reading at 600 nm using the formula:

$$\% {\text{ Fragmented DNA}}\, = \,\left[ {{\mathrm{T}}/\left( {{\mathrm{T}}\, + \,{\mathrm{B}}} \right)} \right]\, \times \,{1}00.$$

where T and B are the OD600 of fragmented DNA in the T and B fractions, respectively.

### Topoisomerases assay

The SB1 and SB2 were evaluated using the human DNA topoisomerase 1 ELISA kit (#MBS289119, MyBioSource, San Diego, CA, USA) and the human DNA topoisomerase II ELISA kit (#EKN48879-96 T, BIOMATIK, Wilmington, DE, USA) to determine their inhibitory activity against TOPO I and TOPO II enzymes. The synthesized SB1 and SB2 were administered to HCT116 and MCF-7 cells at the IC_50_ value. Following the extraction of both treated and untreated cells, TOPO I and TOPO II were measured in accordance with the manufacturer’s instructions. After obtaining a standard curve, the absorbance at 450 nm was measured. The standard curve was used to compute the concentrations of TOPO I and TOPO II. The experiment was performed in triplicate.

### *In silico* studies

Using the “SMILES-generator-checker” program, the Simplified Molecular Input Line Entry System (SMILES) representations for the compounds under study were created. The molecular structures for the ensuing computational investigations were built using these SMILES strings. LigTMap^30^ and Swiss target^31^ Prediction tools used to anticipate the possible protein targets of the compounds under study. ADMET Profiling Using the ADMET lab 2.0 online platform^32^, the properties of absorption, distribution, metabolism, excretion, and toxicity (ADMET) were assessed. To evaluate each molecule’s drug-likeness and safety profile, important pharmacokinetic characteristics were calculated, including LogP, LogS, permeability, plasma protein binding, half-life, and toxicity endpoints (e.g., DILI, Ames test, carcinogenicity).

Additionally, the pathways of compounds SB1 and SB2 were predicted in silico using the Pathway Map tool on the play molecule website (playmolecule.org)^33^. The SMILES strings for SB1 was (O=S(=O)(Nc1ncccn1)c3ccc(/N=C/c2ccccc2O)cc3) and for SB2 was (O=S(=O)(Nc1ncccn1)c3ccc(/N=C/c2cc(Br)ccc2O)cc3) were used in the PathwayMap and the predicted activation or inhibition of pathways by the query compounds were outputted.

The predicted pathways for compounds SB1 and SB2 from PathwayMap were confirmed using the STITCH chemical-chemical interaction database (stitch.embl.de). The SMILES strings were inputted into STITCH chemical and searched against the database using the highest similarity compound score (Sulfadiazine). The pathways predicted by STITCH chemical were compared to the PathwayMap predictions to confirm the results^34^.

### Molecular docking analysis

Molecular docking study was performed to assess the potential affinity of the tested compounds against Carbonic anhydrase XII. Subsequently, our candidates were docked against the potential target Carbonic anhydrase XII (PDB IDs: 8co3)^35^ and The tested compounds were docked against Human DNA topoisomerase II ATPase target site (PDB code: 1ZXN)^36^.

Initially, water molecules were eliminated from the protein complexes, and unnecessary molecules were ignored. The empty valence atoms and disorders were then fixed. PDBQT files were created by minimizing the energy of the protein structures. Each compound’s 2D structure was created with Chem-Bio Draw Ultra16.0 and saved as an SDF file. Protonation and energy minimization were then completed and stored as a PDBQT file, the prepared ligand was docked against Carbonic anhydrase XII active pocket with center (X = − 4.668, Y = 6.338, and Z = 7.419) with grid box size (30*30*30), then the best scoring poses were chosen. Using the rigid methodology, which was carried out with Autodock Vina 1.5.7 software^37^, the ligands were permitted to be flexible while the receptor was kept rigid. Using the Discovery Studio 2024 visualizer, 3D and 2D figures were created based on the docking scores of the best-fitted poses with the active sites^38^.

Protein loops were prepared by excluding water molecules and other non-essential components. Structural disorders and unfilled valence atoms were corrected prior to energy minimization. The optimized protein structure was then saved in PDBQT format^39^. The two-dimensional chemical structures of the compounds investigated were exported as SDF files. Ligand preparation, including protonation and energy minimization, was performed before converting the structures into PDBQT format^40^. Molecular docking study was carried out using AutoDock Vina version 1.5.7. The prepared ligands were docked into the critical binding site of the Human DNA topoisomerase II ATPase target site, and the resulting binding poses were ranked based on their binding affinity scores^37^. The docking scores (affinity energy) of the best-fitted poses with the active sites were recorded, and 3D and 2D figures were generated using Discovery Studio 2024 visualizer^38^.

### Statistical analysis

All experiments were performed in triplicates, and the results are expressed as mean ± standard deviation (SD). Statistical analyses were carried out using GraphPad Prism version 9 (GraphPad Software, San Diego, CA, USA). Differences among groups were evaluated using one-way analysis of variance (ANOVA). A *p*-value of less than 0.05 (*p* < 0.05) was considered statistically significant.

## Results

### Characterization of the synthesized Schiff base compounds

The IR spectra of SB1 and SB2 showed notable absorption bands that corresponded to azomethine (C=N) at 1619 cm⁻^1^, N–H (3164–3123 cm⁻^1^), and asymmetric SO₂ stretching at 1339–1336 cm⁻^1^. These bands confirmed successful Schiff base formation and retention of sulfonamide functionality. Condensation was further confirmed by the ^1^H NMR spectra (DMSO-d₆, 400 MHz), which showed singlets at δ 8.9–9.0 ppm corresponding to the azomethine proton (CH=N). Phenolic OH protons (exchangeable with D₂O) were identified as the source of signals at δ ~ 12.5 ppm, indicating intramolecular hydrogen bonding. The presence of aromatic protons in the δ 6.5–8.6 ppm region was consistent with heterocyclic and substituted phenyl rings. The NH proton disappeared upon D₂O exchange after being detected at δ 6.1 ppm. The suggested molecular formulae and high purity of synthesized compounds were confirmed by the elemental analysis data, which showed good agreement with calculated values. ^13^C NMR (DMSO-d₆, 100.6 MHz) spectra in both compounds showed that the azomethine carbon (C=N) was identified as the source of the resonance seen at about δ 172 ppm, suggesting successful Schiff base condensation. Carbons bound to heteroatoms, such as phenolic (C–OH), heteroaromatic (C–N), and aromatic carbons next to the sulfonamide (C–SO₂) group, are represented by the cluster of signals that emerge between δ 152 and 166 ppm. Multiple resonances within the δ 110–140 ppm region support the conjugated π-system’s preservation and are consistent with aromatic and heteroaromatic carbons from the phenyl rings and the nitrogen-containing heterocycle.

### Antiproliferative activity

The activity of the synthesized Schiff base compounds, SB1 and SB2, was estimated for the different human cancer cell lines; MCF-7, HCT116, A549, T24, and HepG2, normalized to normal lung epithelial cell line WI-38 by the MTT assay.

SB1 compound had the highest antiproliferative activity for the MCF-7 (66.27 ± 1.14 µg/mL), followed by HCT116 (90.01 ± 0.12 µg/mL), A549 cells (91.5 ± 2.06 µg/mL), T24 (192.28 ± 1.12 µg/mL) and HepG-2 cells (240.08 ± 3.83 µg/mL) compared to the WI-38 cells (329.61 ± 3.2 µg/mL). From the IC_50_ values, the selectivity index of SB1 for MCF-7, HCT116, A549, T24 and HepG-2 cells were 4.97, 3.66, 3.6, 1.71, and 1.37 respectively, paralleled to the WI-38 cells. Furthermore, the synthesized compound SB2 had the highest antiproliferative activity for the HCT116 (48.65 ± 2.12 µg/mL), followed by MCF-7 (59.61 ± 1.59 µg/mL), A549 cells (68.56 ± 1.6 µg/mL), T24 (216.13 ± 0.7 µg/mL) and HepG-2 cells (120.53 ± 2.62 µg/mL) compared to the WI-38 cells (235.41 ± 1.83 µg/mL). From the IC_50_ values, the selectivity index of SB2 for MCF-7, HCT116, A549, T24 and HepG-2 cells were 3.95, 4.84, 3.43, 1.09 and 1.95 respectively, paralleled to the WI-38 cells. Fluorouracil (5-FU, 5-fluorouracil) was used as a control drug. The IC_50_ value of the tested compounds (µg/mL) and its selectivity index against WI-38 cells was listed in (Table 1).

**Table 1:** The IC₅₀ and selectivity index values obtained for the SB1 and SB2 compounds.

Cancer cells											Normal cells
Compound	HCT116		MCF-7		HepG2		A549		T24		WI-38
	IC₅₀ (µg/mL)	SI	IC₅₀ (µg/mL)	SI	IC₅₀ (µg/mL)	SI	IC₅₀ (µg/mL)	SI	IC₅₀ (µg/mL)	SI	IC₅₀ (µg/mL)
SB1	90.01 ± 0.12	3.66	66.27 ± 1.14	4.97	240.08 ± 3.83	1.37	91.5 ± 2.06	3.6	192.28 ± 1.12	1.71	329.61 ± 3.2
SB2	48.65 ± 2.12	4.84	59.61 ± 1.59	3.95	120.53 ± 2.62	1.95	68.56 ± 1.60	3.43	216.13 ± 0.70	1.09	235.41 ± 1.83
5-Fu	3.6 ± 0.33	3.72	5.13 ± 0.39	2.61	7.06 ± 0.6	1.89	6.94 ± 0.61	1.93	6.68 ± 0.22	2.0	13.4 ± 0.28

**Fig. 1:**
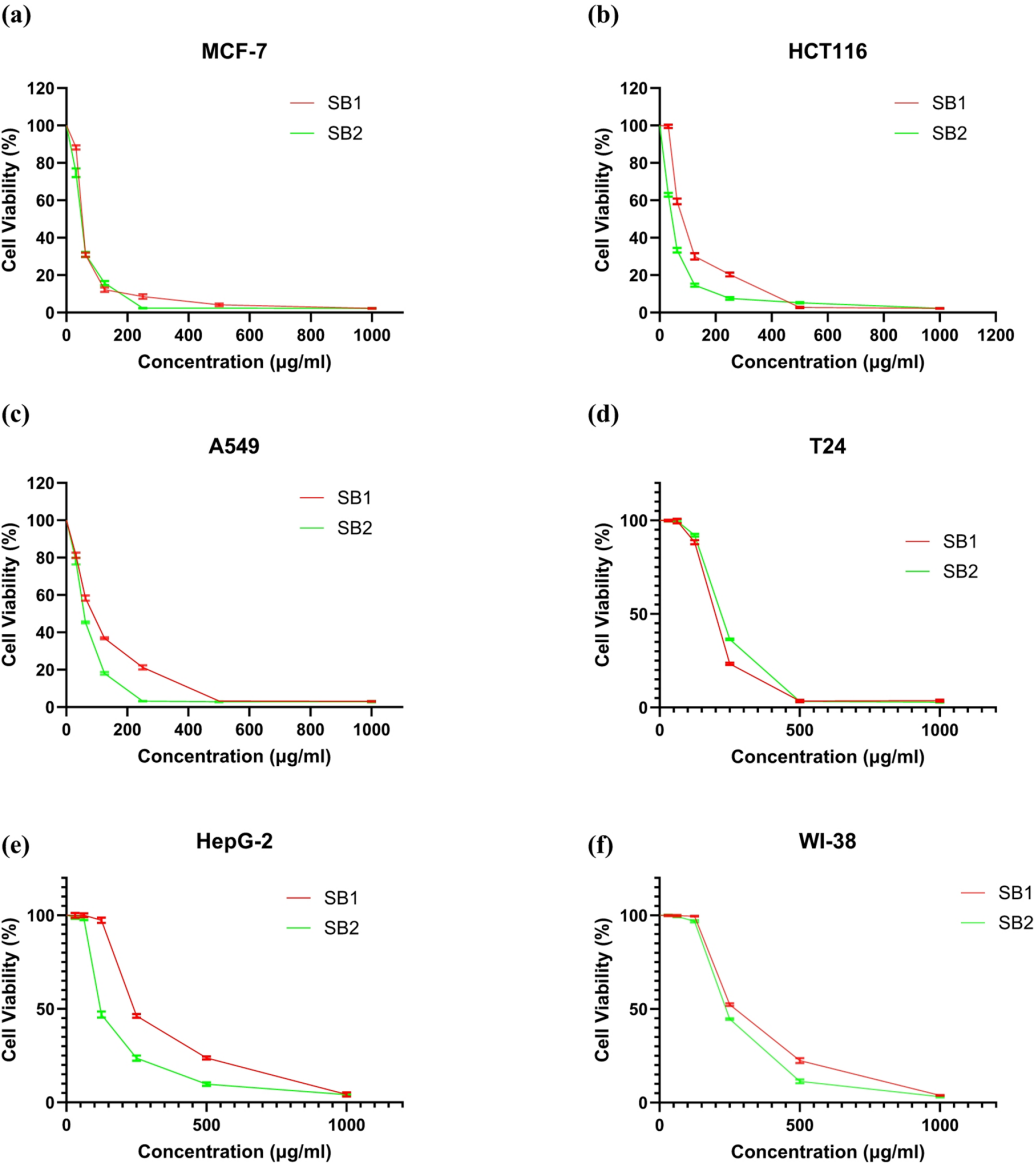
Cytotoxic activity of SB1 and SB2 against various tumor cell lines. Cell viability was assessed in breast carcinoma (MCF-7) (**a**), colon carcinoma (HCT116) (**b**), lung carcinoma (A549) (**c**), urinary bladder carcinoma (T24) (**d**), and hepatocellular carcinoma (HepG2) (**e**). Normal human lung epithelial cells (WI-38) were included as a control (**f**). Data are presented as mean ± SD.

All investigated cancer cell lines showed moderate, concentration-dependent antiproliferative effects from SB1 and SB2, according to the results. Interestingly, SB2’s lower IC₅₀ values in MCF-7, HCT116, A549, and HepG2 cells showed that it was generally more cytotoxic than SB1. The most affected cell lines were MCF-7 and HCT116, indicating that SB1 and SB2 may have selective effects on particular tumor types. On the other hand, a degree of selectivity of the compounds toward malignant cells over normal cells was indicated by the relatively larger IC₅₀ values displayed by the normal WI-38 cells. This selectivity reduces the possibility of damage to healthy tissues, which is a desired characteristic for anticancer drugs (Fig. 1).

### Apoptosis analysis by annexin V-FITC/PI staining

**Fig. 2:**
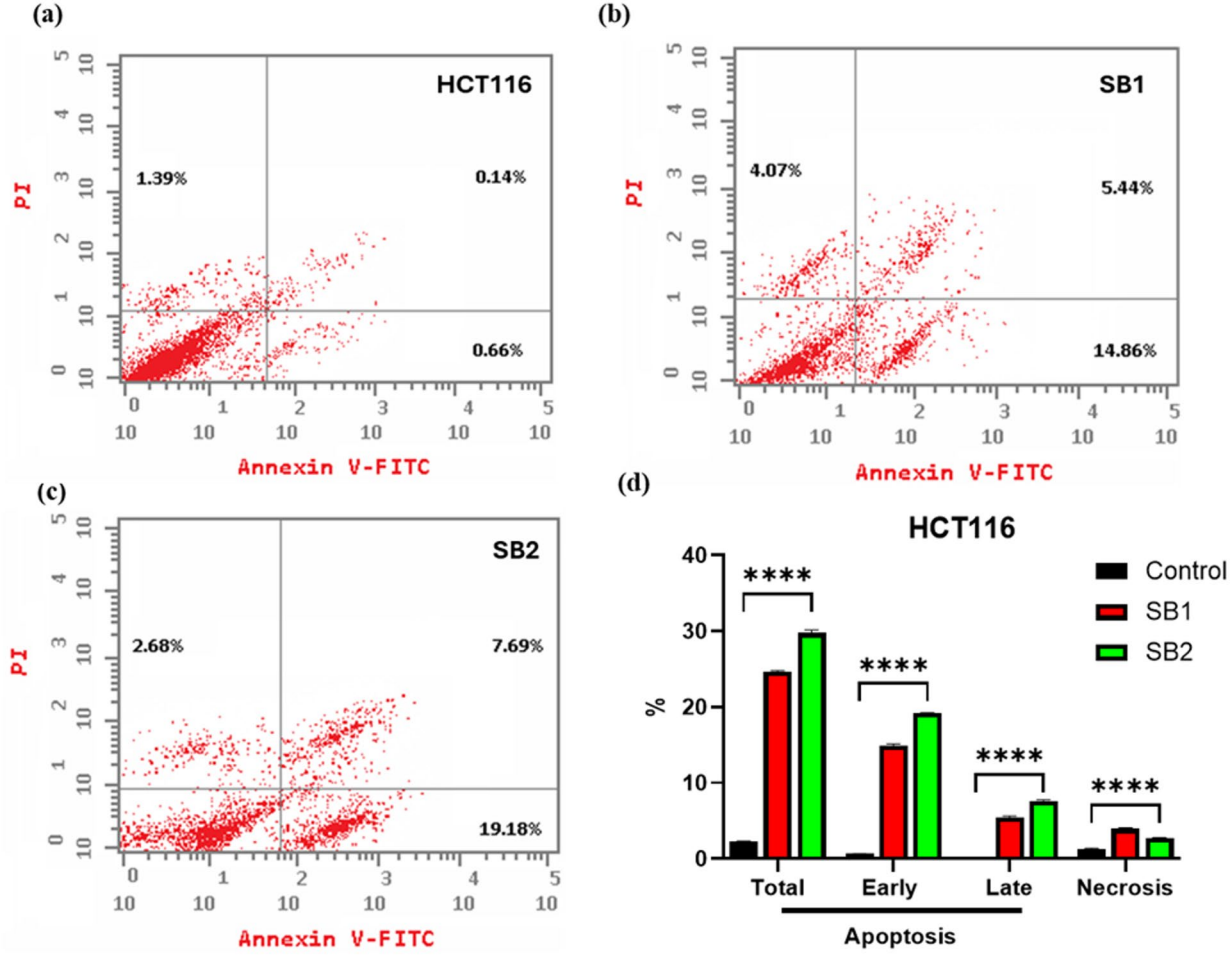
Apoptosis analysis by flow cytometry with Annexin V-FITC/PI dual stains of the HCT116 cells. The cells were treated with IC_50_ of SB1 and SB2, the apoptosis was measured after 48 h of incubation. Untreated HCT116 cells (control) (**a**) Apoptotic analysis of SB1 (**b**) and after SB2 treatment (**c**), and overall quantitative results of apoptosis (**d**). The results were statistically significant compared to the untreated cells controls (*p* < 0.05).

**Fig. 3:**
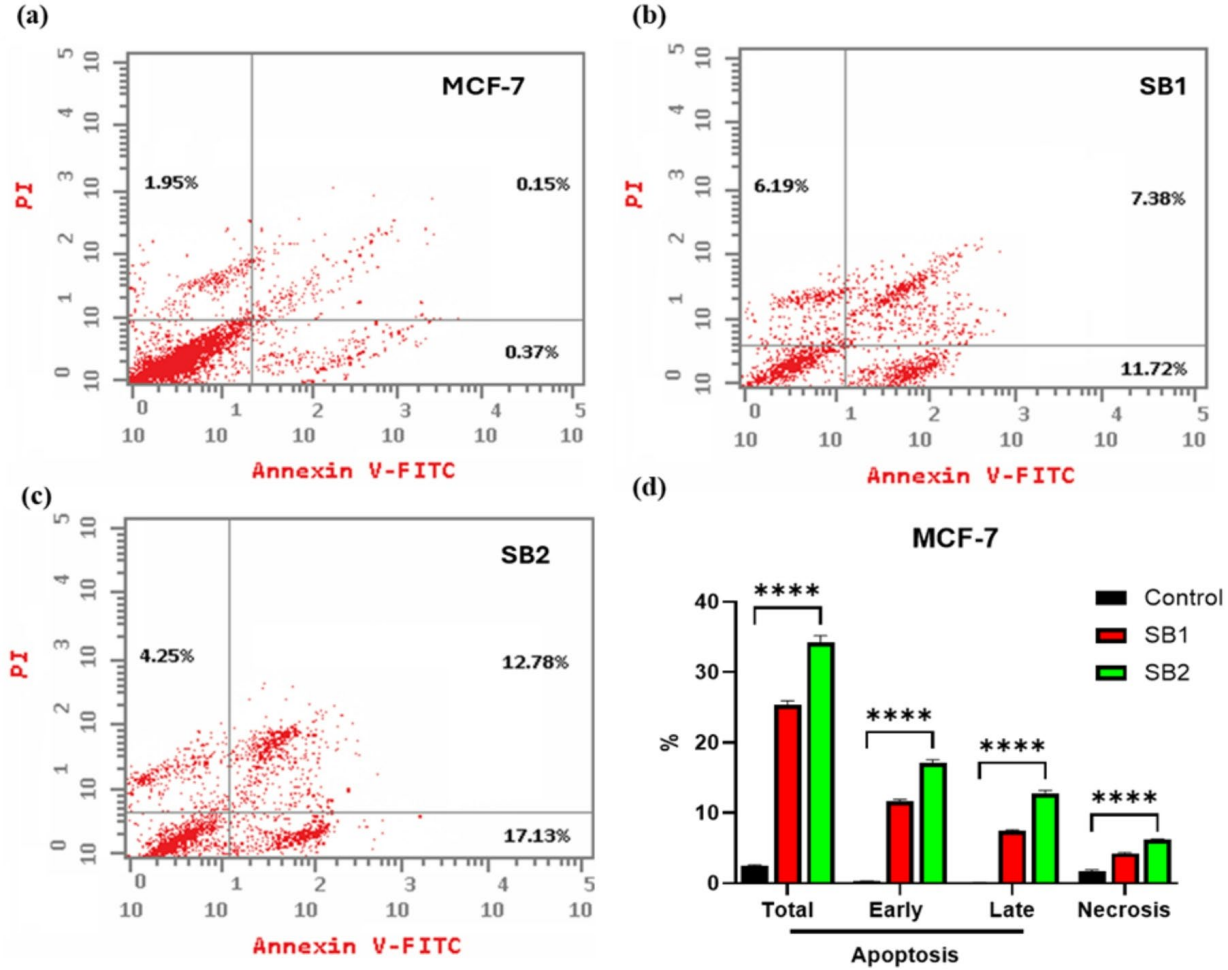
Apoptosis analysis by flow cytometry with Annexin V-FITC/PI dual stains of the MCF-7 cells. The cells were treated with IC_50_ of SB1 and SB2, the apoptosis was measured after 48 h of incubation. Untreated MCF-7 cells (control) (**a**) Apoptotic analysis of SB1 (**b**) and after SB2 treatment (**c**), and overall quantitative results of apoptosis (**d**). The results were statistically significant compared to the untreated cells controls (*p* < 0.05).

Annexin V-PI staining was used to evaluate apoptosis in HCT116 and MCF-7 cells following treatment with SB1 and SB2. The obtained results showed a considerable induction of the cells on the various apoptotic processes “early apoptosis, late apoptosis” (Figs. 2, 3). SB1 caused 24.37, 14.86, and 5.44% of the total HCT116 cells to undergo early and late apoptosis, while SB2 caused 29.55, 19.18, and 7.69% of the cells to undergo these processes. Nonetheless, the untreated HCT116 control cells’ apoptosis ratio was roughly 2.19%, 0.66%, and 0.14%, respectively, in response to SB1, and for SB2 was 29.55, 19.18 and 7.69%. Nevertheless, the proportion of control cell apoptosis (untreated HCT116) was about 2.19%, 0.66%, and 0.14%, respectively. Thus, with SB1 treatment, the total apoptosis was increased by 11 folds, and with SB2 increased by 13 folds related to control, ensuring the significant biochemical effect of SB2 than SB1 in triggering the apoptotic machinery in cells. Additionally, compared to the untreated cells (1.39%), the ratio of necrosis of normal HCT116 cells increased to almost 2.9% in the presence of SB1 and 1.9% for SB2, confirming the cytotoxicity and specificity of the synthesized compounds, the results were shown in Fig. 3.

Moreover, the percentage of MCF-7 cells in total early and late apoptosis were 25.29, 11.72 and 7.38%, respectively, in response to SB1, and for SB2 was 34.16, 17.13 and 12.78%. However, the ratio of apoptosis of control cells (untreated HCT116) was about 2.47, 0.37 and 0.15% respectively. Thus, with SB1 treatment, the total apoptosis was increased by 10 folds, and with SB2 increased by 14 folds related to control, ensuring the significant biochemical effect of SB2 than SB1 in triggering the apoptotic machinery of cells. Additionally, compared to the untreated cells (1.95%), the ratio of necrosis of normal MCF-7 cells increased to approximately 3.2% in the presence of SB1 and 2.2% for SB2, ensuring the cytotoxicity and specificity of the synthesized compounds. as shown in (Fig. 3).

### Effect of Schiff bases on apoptotic protein expression

**Fig. 4:**
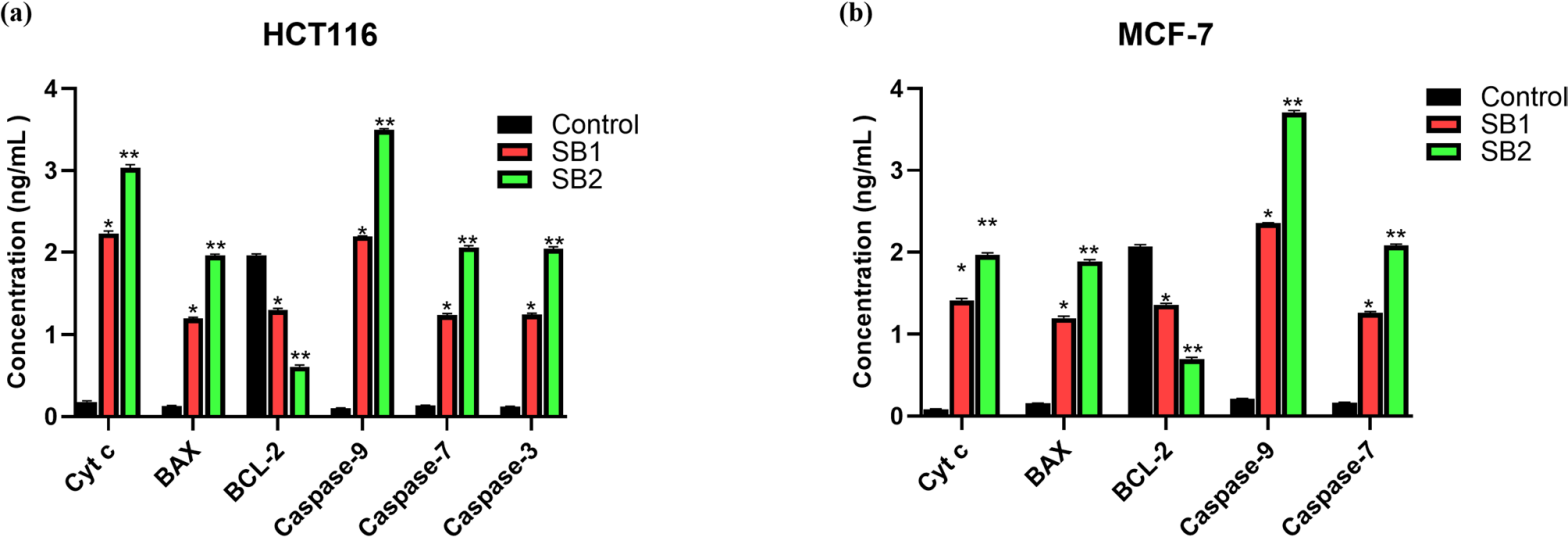
Quantitative analysis of apoptotic protein expression following treatment with SB1 and SB2 in HCT116 (**a**) and MCF-7 cells (**b**). Data are presented as mean ± SD. *Significant difference as compared to the control (*p* < 0.05).

Treatment of SB1 and SB2 markedly altered apoptotic markers in both HCT116 and MCF-7 cell lines (Fig. 4). Cytochrome c (Cyt c) concentrations were significantly increased in both cell types. Cyt c levels in HCT116 cells increased from 0.179 ± 0.0127 ng/mL in control cells to 2.233 ± 0.0269 ng/mL and 3.037 ± 0.0361 ng/mL after SB1 and SB2 treatment, respectively. In MCF-7 cells, Cyt c levels increased from 0.082 ± 0.0049 ng/mL in control cells to 1.416 ± 0.0219 ng/mL and 1.964 ± 0.0283 ng/mL following treatment.

A similar trend was noted for the pro-apoptotic protein Bax, which was markedly increased in both cell lines. Bax levels in HCT116 cells increased from 0.132 ± 0.0026 ng/mL to 1.20 ± 0.0129 ng/mL and 1.96 ± 0.0183 ng/mL after treatment with SB1 and SB2, respectively. Bax levels in MCF-7 cells increased from 0.154 ± 0.0029 ng/mL to 1.20 ± 0.0208 ng/mL and 1.89 ± 0.0171 ng/mL respectively. Conversely, the anti-apoptotic protein Bcl-2 was uniformly downregulated in both cell lines. Bcl-2 levels in HCT116 cells diminished from 1.96 ± 0.0208 ng/mL in control cells to 1.30 ± 0.0208 ng/mL and 0.61 ± 0.0171 ng/mL after SB1 and SB2 treatment, respectively. Bcl-2 levels in MCF-7 cells decreased from 2.07 ± 0.0208 ng/mL to 1.36 ± 0.0171 ng/mL and to 0.70 ± 0.0171 ng/mL respectively.

In accordance with the activation of the intrinsic apoptotic pathway, caspase-9 levels were elevated in both cell lines. Caspase-9 levels in HCT116 cells increased from 0.100 ± 0.0035 ng/mL in control cells to 2.198 ± 0.0035 ng/mL and 3.498 ± 0.0141 ng/mL after treatment with SB1 and SB2, respectively. In MCF-7 cells, caspase-9 levels increased from 0.212 ± 0.0035 ng/mL to 2.354 ± 0.0050 ng/mL and subsequently to 3.704 ± 0.0283 ng/mL. Caspase-7 levels exhibited a similar pattern, rising from 0.135 ± 0.0026 ng/mL to 1.24 ± 0.0171 ng/mL and 2.06 ± 0.0208 ng/mL in HCT116 cells, and from 0.165 ± 0.0029 ng/mL to 1.26 ± 0.0171 ng/mL and 2.08 ± 0.0171 ng/mL in MCF-7 cells. Caspase-3 activity exhibited a significant rise exclusively in HCT116 cells, escalating from 0.122 ± 0.0028 ng/mL in control cells to 1.248 ± 0.0141 ng/mL and 2.041 ± 0.0276 ng/mL after SB1 and SB2 treatment, respectively. These data demonstrate that SB1 and SB2 promote apoptosis by modulating mitochondrial-associated apoptotic pathways, as seen by elevated levels of Cyt c, Bax, and caspases, alongside reduced Bcl-2 levels. The results were consistently more significant with SB2, indicating superior pro-apoptotic efficacy relative to SB1.

### Determination of intracellular reactive oxygen species (ROS) production

**Fig. 5:**
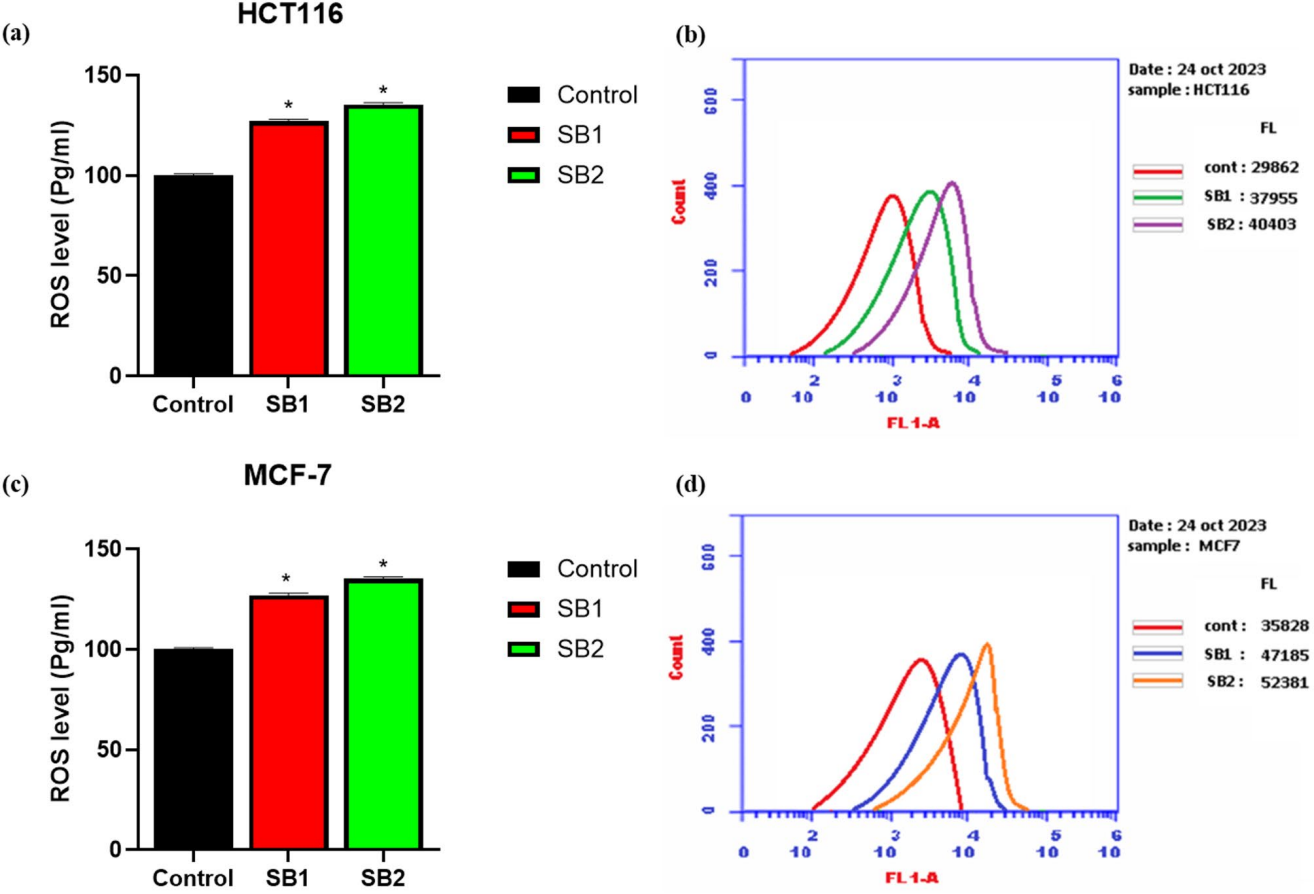
Generation of ROS of HCT116 (**a**, **b**) and MCF-7 (**c**, **d**) cells in response SB1 and SB2 treatment. *Significant difference as compared to the control (*p* < 0.05).

The generation of ROS has been widely used as a marker for a variety of biological processes, including apoptosis, DNA damage, and inflammation. The influence of SB1 and SB2 on generating the ROS in HCT116 and MCF-7 cells was assessed. The ROS level of treated HCT116 and MCF-7 cells at their IC_50_ concentration was significantly elevated upon exposure to the Schiff base compounds, as shown in Fig. 5. A flow cytometry histogram with three overlay curves that represent several samples is displayed in the picture. The “FL 1-A” label on the x-axis seems to indicate fluorescence intensity, while the y-axis shows the number of cells or events. Both Schiff base compounds, SB1 and SB2, considerably raised intracellular ROS levels in HCT116 and MCF-7 cells in comparison to their respective untreated controls. For MCF-7 cells, SB1 and SB2 increased ROS generation to 131.7% and 146.2%, respectively, whereas SB1 increased ROS levels in HCT116 cells to 127.1% and SB2 to 135.3% over the control (without treatment).

### DPA assay test

**Fig. 6:**
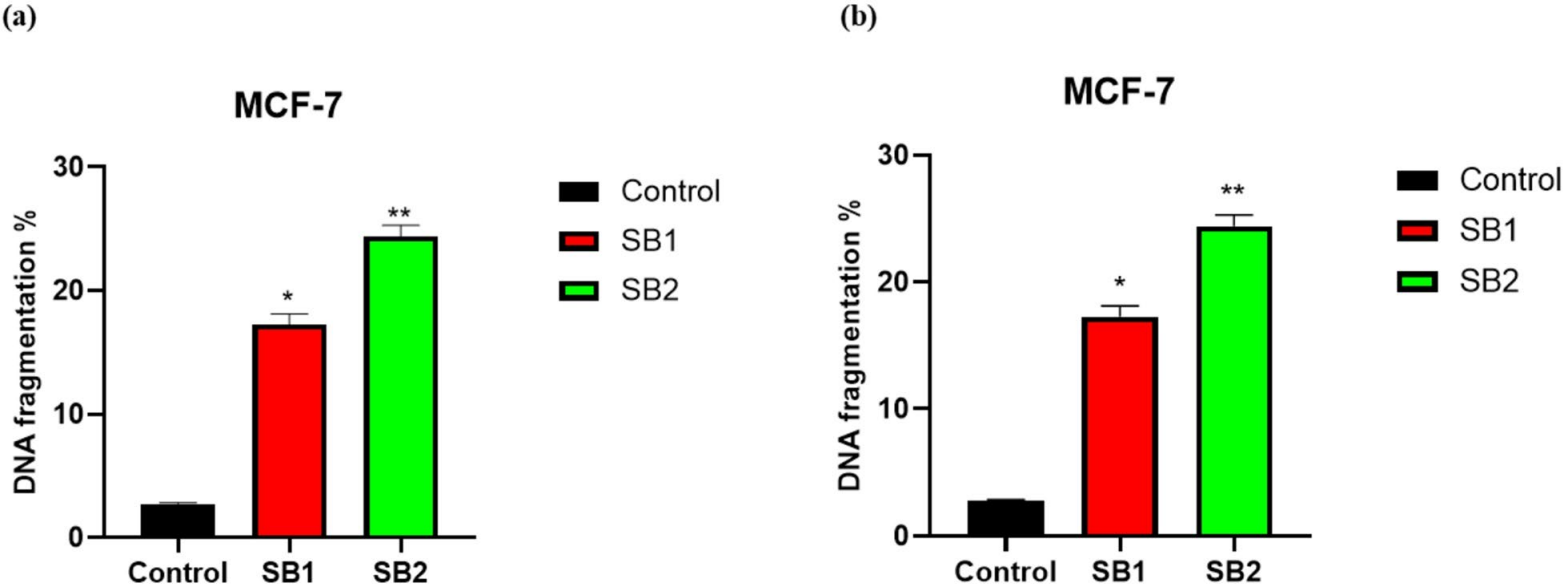
DNA fragmentation on HCT116 and MCF-7 cells after treatment with SB1and SB2. untreated cells used as control. *Significant difference as compared to the control (*p* < 0.05).

The diphenylamine assay is a highly effective approach for evaluating apoptosis because it determines the percentage of DNA fragmentation into oligosomal-sized fragments. The amount of soluble DNA released from apoptotic nuclei into the cytoplasm is a quantitative indicator of cellular response. Another feature of the diphenylamine assay is that it can identify apoptotic DNA fragmentation in both adhering and floating cells after treatment with chemotherapeutic or other drugs. Figure 6, shows the degree of DNA fragmentation caused by SB1 and SB2 treatment on HCT116 and MCF-7 cells, indicating their pro-apoptotic potential and markedly increased DNA fragmentation; however, SB2 effect was more noticeable in HCT116 and MCF-7 cells than SB1 when compared to the control (untreated cells). In particular, DNA fragmentation on HCT116 treated with SB1 and SB2 was 18.4 and 25.07% respectively when compared to the control cells (2.52), meanwhile, the DNA fragmentation on MCF-7 cells treated with SB1 and SB2 was 17.25 and 24.42%, respectively, and the control value was 2.74. These results are consistent with previous MTT assay findings and support the idea that SB2 induces cell death through DNA damage-mediated apoptosis. This implies that SB2 may exhibit greater anticancer potential than SB1.

### Inhibition of TOPO I and II enzymes

**Fig. 7:**
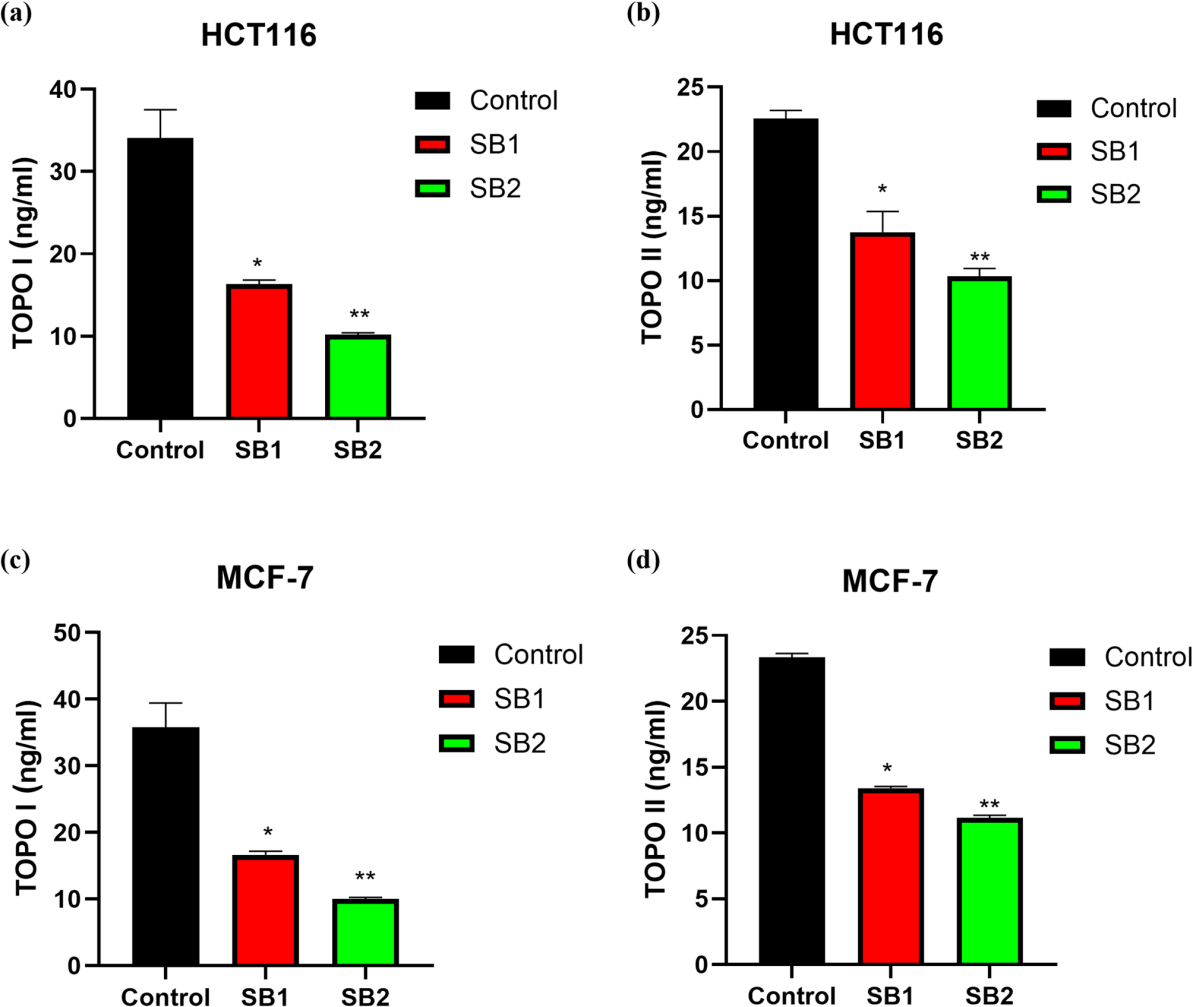
TOPO I and TOPO II on HCT116 and MCF-7 cells after treatment with SB1and SB2. Activity of TOPO I and TOPO II on HCT116 (**a**, **b**), MCF-7 (**c**, **d**). *Significant difference as compared to the control (*p* < 0.05).

To assess the activity of TOPO I and TOPO II enzymes, the IC_50_ of the synthesized compounds applied to HCT116 and MCF-7 cancer cells. Figure 7 illustrates how, in comparison to the untreated cells, the SB1 and SB2 derivatives significantly (*p* < 0.01) reduced the activity of TOPO I and TOPO II enzymes in HCT116 and MCF-7 cells. SB1 and SB2 both significantly reduced TOPO I and TOPO II activity when compared to control (untreated cells). A significant inhibition of TOPO1 activity on HCT116 after treatment with SB1 and SB2 was 52.07 and 69.98% when compared to control (*P* < 0.05). Meanwhile, the TOPO1 activity on MCF-7 inhibited by SB1 and SB2 was 39.01 and 54.12% respectively, SB2 demonstrates significantly more inhibitory effects on both TOPO1 compared to SB1 across HCT116 and MCF-7 cells.

### In silico *studies*

#### Targets prediction

**Fig. 8:**
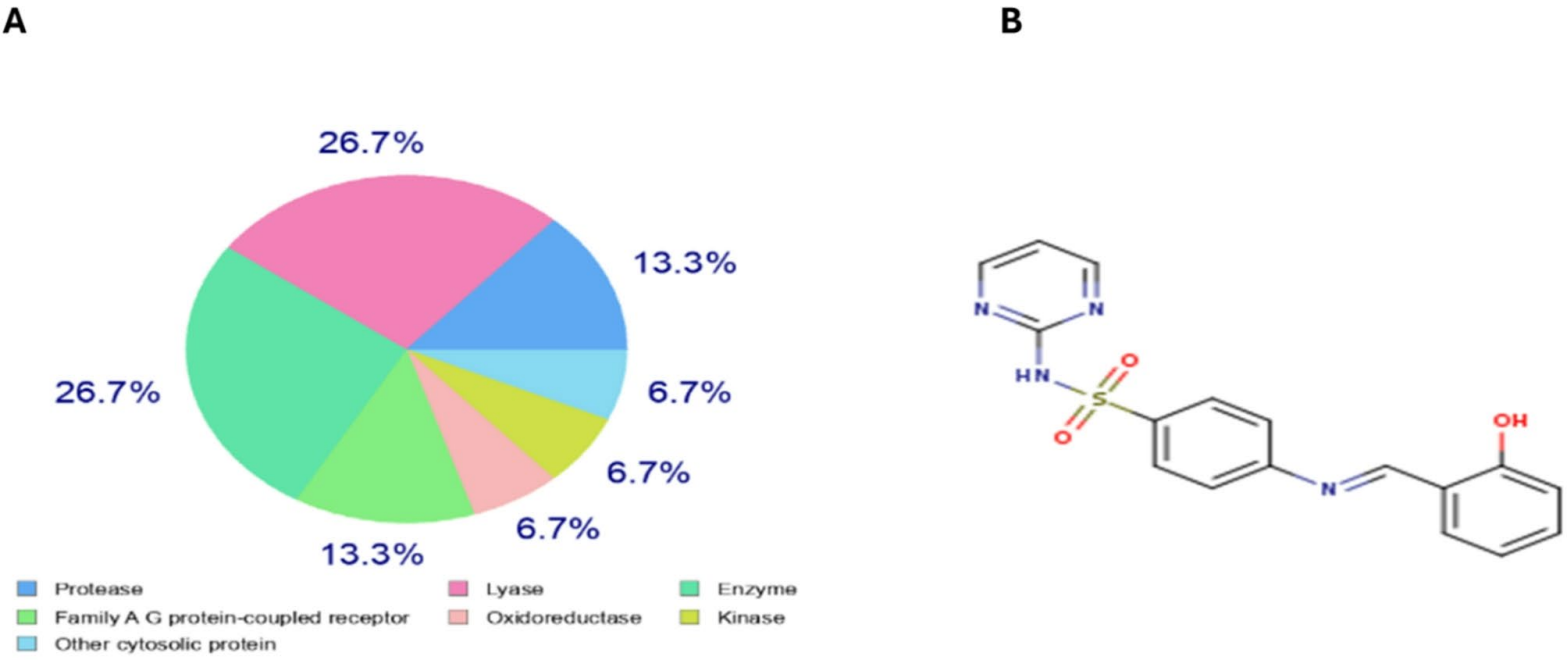
(**A**) Pie chart represented the target classification for SB1, represent equal prediction for lyase protein targets and family A G protein-coupled receptor. (**B**) Structure from smiles code generator for SB1.

**Fig. 9:**
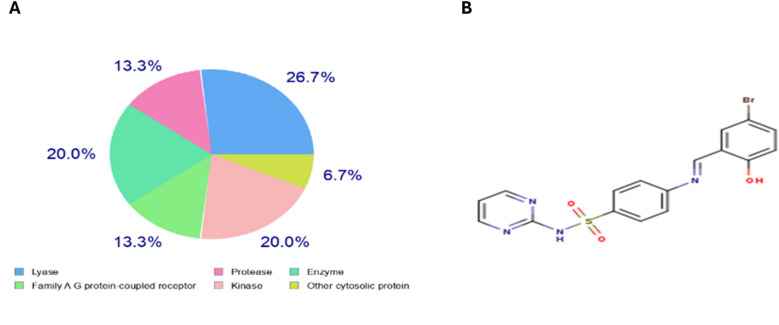
(**A**) Pie chart represented the target classification for SB2, represent higher prediction for lyase protein targets more than family A G protein-coupled receptor targets. (**B**) Structure from smiles code generator for SB2.

Key enzymes like carbonic anhydrases (CA1, CA2, CA9, CA12), bone morphogenetic protein 1 (BMP1), kinases like BRAF and CDK2/Cyclin A, and G protein-coupled receptors like endothelin receptor ET-B (EDNRB) and adenosine A1 receptor (ADORA1) were among the 18 and 11 predicted targets, respectively, that SwissTargetPrediction and LigTMap analysis of SB1(Fig. 8) had identified With a comparable variety of proteins found, such as carbonic anhydrases, BMP1, BRAF, EGFR, and SGK1, as well as targets like 15-hydroxyprostaglandin dehydrogenase and the Pregnane X receptor, SB2 (Fig. 9) had a somewhat better affinity for lyase targets. According to ADMET profile, both compounds have poor aqueous solubility (LogS: − 3.536 and − 4.096), low intestine absorption (HIA: 0.021 and 0.006), poor membrane permeability (Caco-2: − 5.417 and − 5.079), and intermediate lipophilicity (LogP: 2.916 and 3.725 for SB1 and SB2, respectively).

Both compounds showed short half-lives (0.188 and 0.133 h) and high plasma protein binding (97.69% and 98.35%). They also had minimal mutagenic potential (Ames: 0.013 and 0.01) and a high predicted risk of drug-induced liver injury (DILI: 0.993 and 0.992). Interestingly, SB2 had a high level of matrix metalloproteinase inhibition (SR-MMP: 0.837), indicating possible anti-metastatic qualities. According to the Lipinski, Pfizer, and Golden Triangle guidelines, both compounds were considered drug-like; however, SB2 did not satisfy the GSK requirements. Although the compounds showed multi-target promise overall, their pharmacokinetic features had limits that call for additional improvement.

#### Pathway prediction

**Fig. 10:**
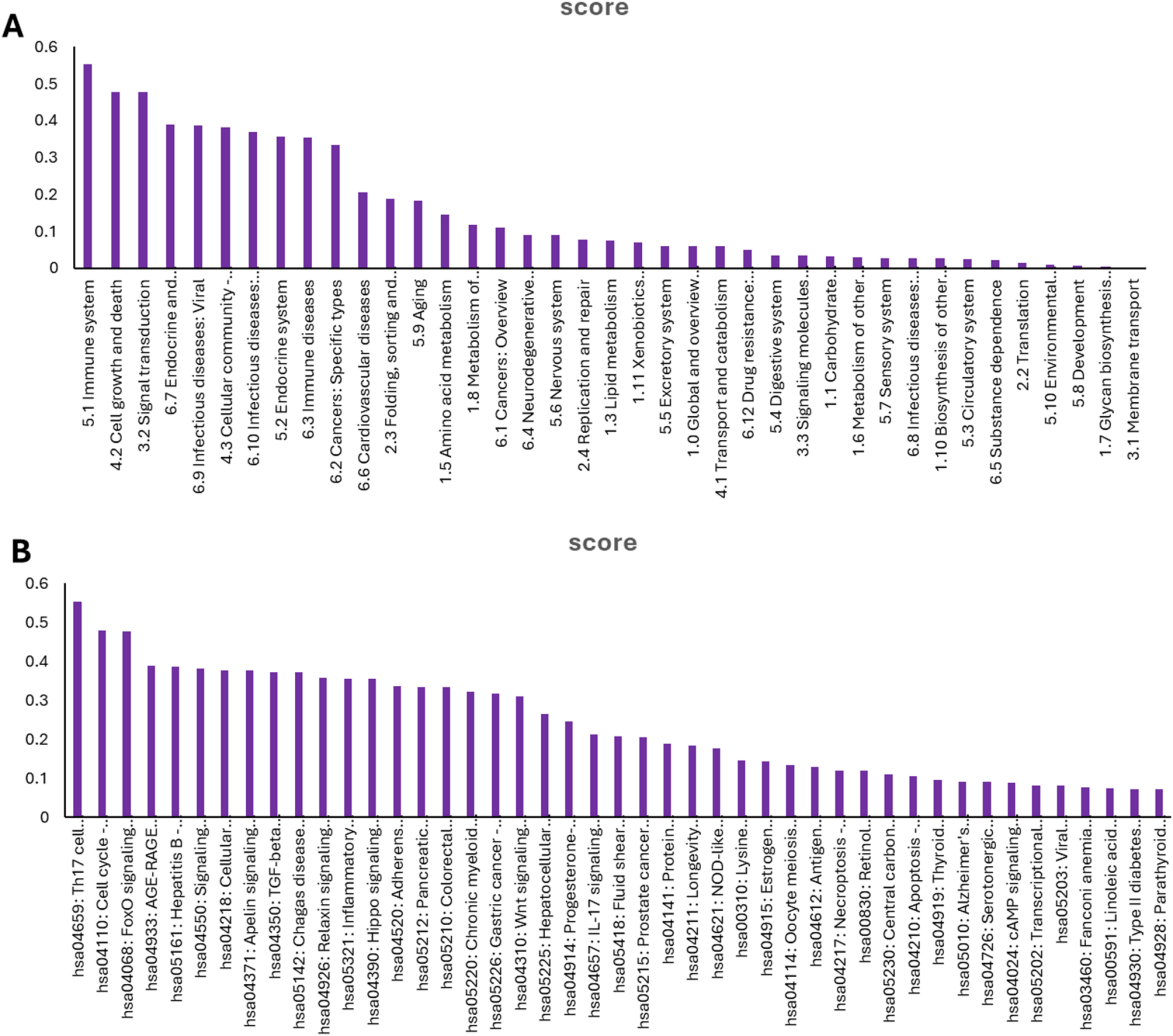
(**A**) Pathway classes integrated in molecule SB1. (The score indicates the likelihood of association between the pathway and the ligand, ranging from 0 to 1, where 1 signifies a high probability of association. (**B**) The specific pathway for compound SB1.

**Fig. 11:**
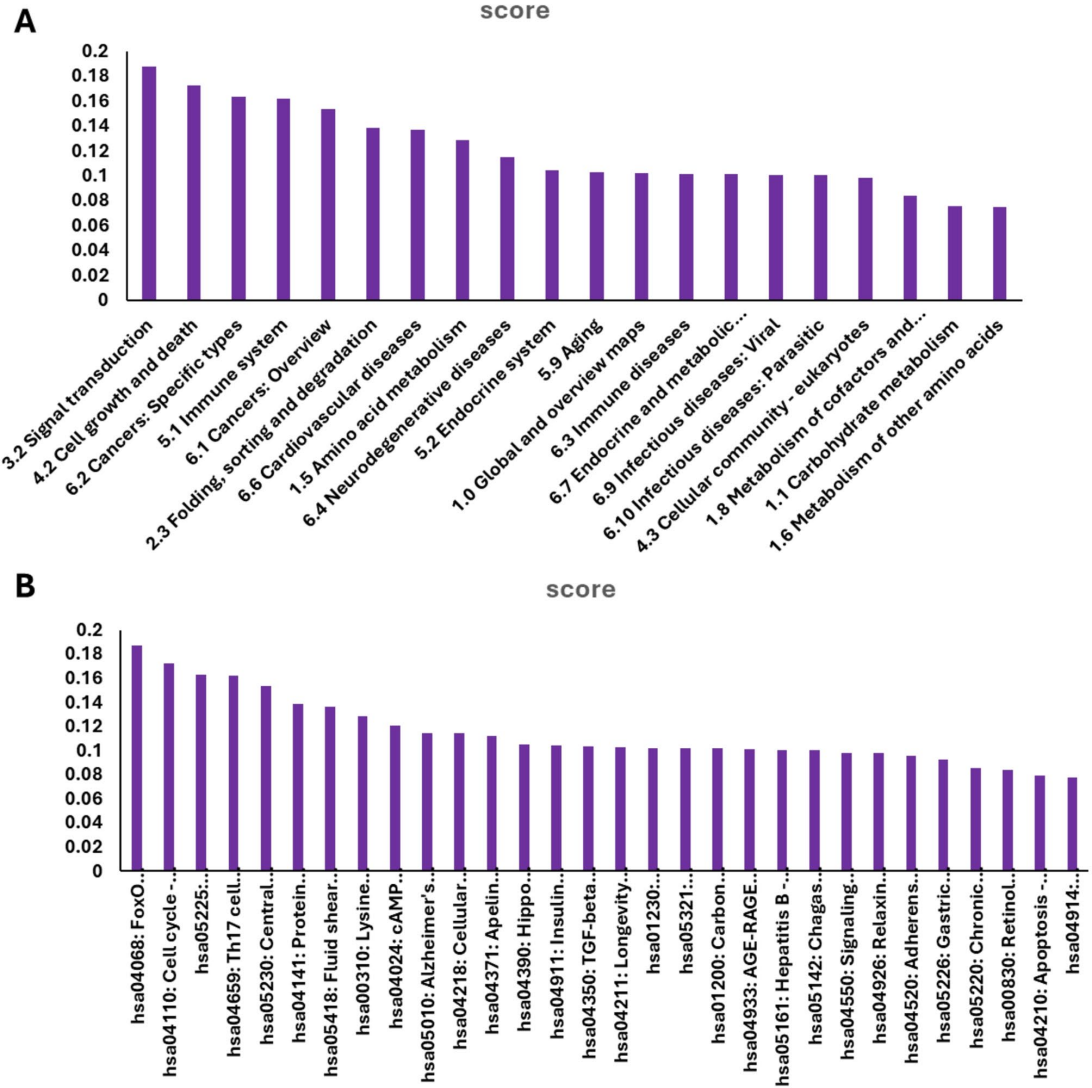
(**A**) pathway classes integrated in molecule SB2. (The score indicates the likelihood of association between the pathway and the ligand, ranging from 0 to 1, where 1 signifies a high probability of association. (**B**) The specific pathway for compound SB2.

The two candidate compounds in silico assessment showed that they have a wide range of possible targets and varying affinities for various biological pathways. Interestingly, SB2 showed a high level of matrix metalloproteinase inhibition (SR-MMP score of 0.837), which may indicate an anti-metastatic effect. These results were further corroborated by STITCH pathway mapping (Fig. 10 and Fig. 11), which connected SB1 to hepatic enzymes including NAT2, CYP2C8, and CYP2C9, validating its known metabolic profile. Overall, the convergence of findings from network-based and ligand-based analyses increases the trust in the anticipated biological activities of these compounds and pinpoints important pathways that could be therapeutically targeted, despite pharmacokinetic constraints that call for additional structural optimization.

### Molecular docking study

The tested compounds were docked against Carbonic anhydrase XII to evaluate their possible binding affinities. The results of the top-screened poses are illustrated in Table 2.

**Fig. 12:**
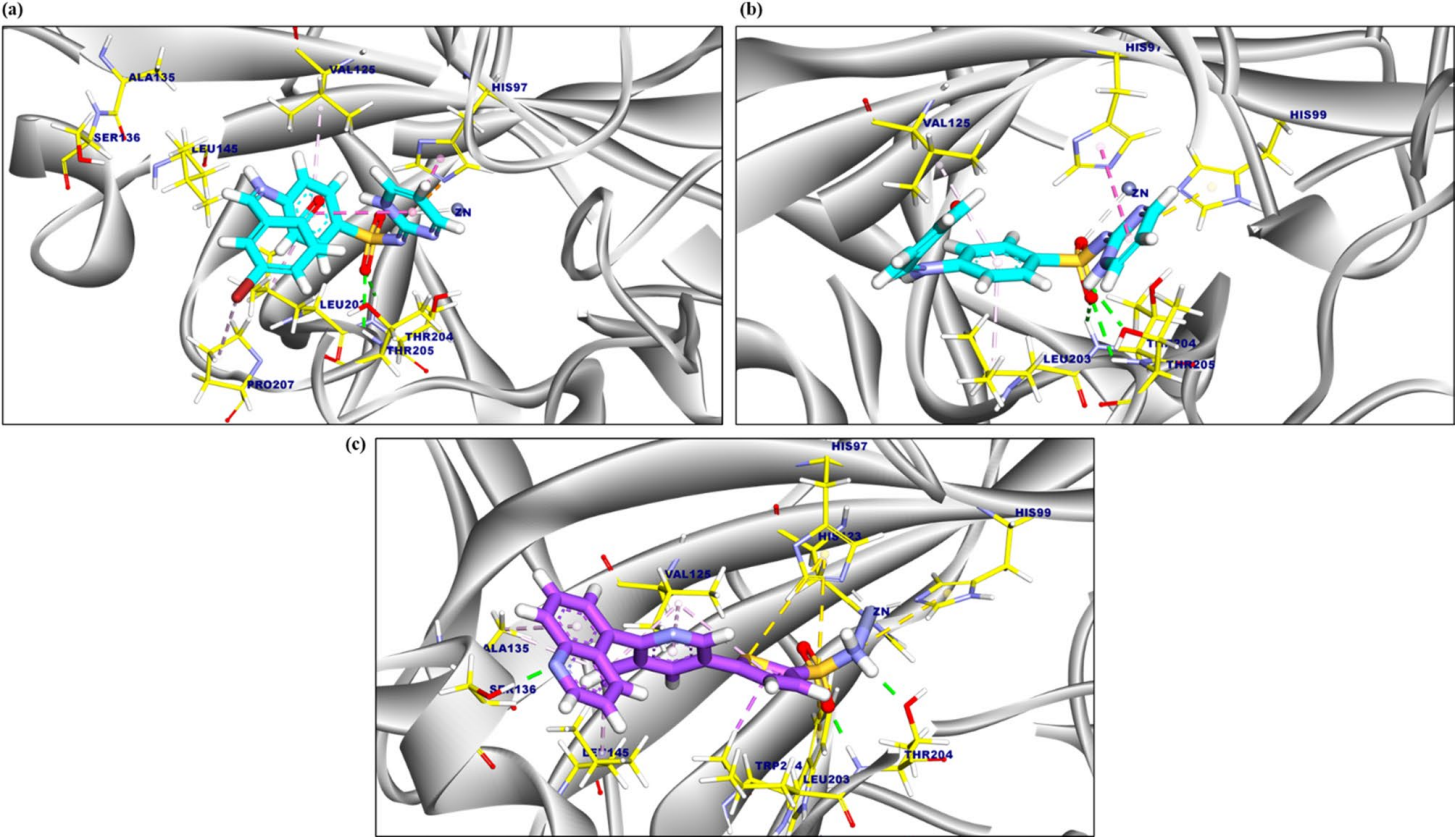
(**A**) 3D figure of the proposed binding mode of SB1 against Carbonic anhydrase XII, amino acids coloured yellow and the ligand coloured turquoise. (**B**) 3D figure of the proposed binding mode of SB2 against Carbonic anhydrase XII , amino acids colored yellow, and the ligand-colored turquoise. (**C**) 3D figure of the proposed binding mode of the co-crystallized ligand complexed with Carbonic anhydrase XII, amino acids colored yellow, and the co-crystallized ligand colored purple.

SB1’s binding mode demonstrated a binding energy of − 6.12 kcal/mol. Against Carbonic anhydrase XII. SB1 interacted with His97, Pro207, Leu203 and Val125, by four hydrophobic π-π, π-anion, and π-alkyl interactions. Moreover, SB1 formed two hydrogen bonds and one metal ion interaction with Thr204, Thr205, and Zn301 with distances of 3.02, 2.03, 2.17, and 2.66 Å, respectively (Fig. 12a). Additionally, the binding affinity of SB2 was − 7.95 kcal/mol. against Carbonic anhydrase XII. Which formed four hydrophobic π-π, π-anion, and π-alkyl interactions with His97, His99, Leu203 and Val125, additionally, the interaction was supported by three strong hydrogen bonds with Thr204, and Thr205, with distances of 2.94, 2.24 and 2.57 Å, respectively (Fig. 12b). The co-crystalized ligand (sulfonamide derivative) complexed with Carbonic anhydrase XII exhibited an affinity score of − 6.76 kcal/mol, it formed eleven π-sulfur, π-sigma and π-alkyl interactions and three hydrogen bonds with Thr204, and Ser136, with distances of 1.93, and 1.82 Å (Fig. 12c).

**Fig. 13:**
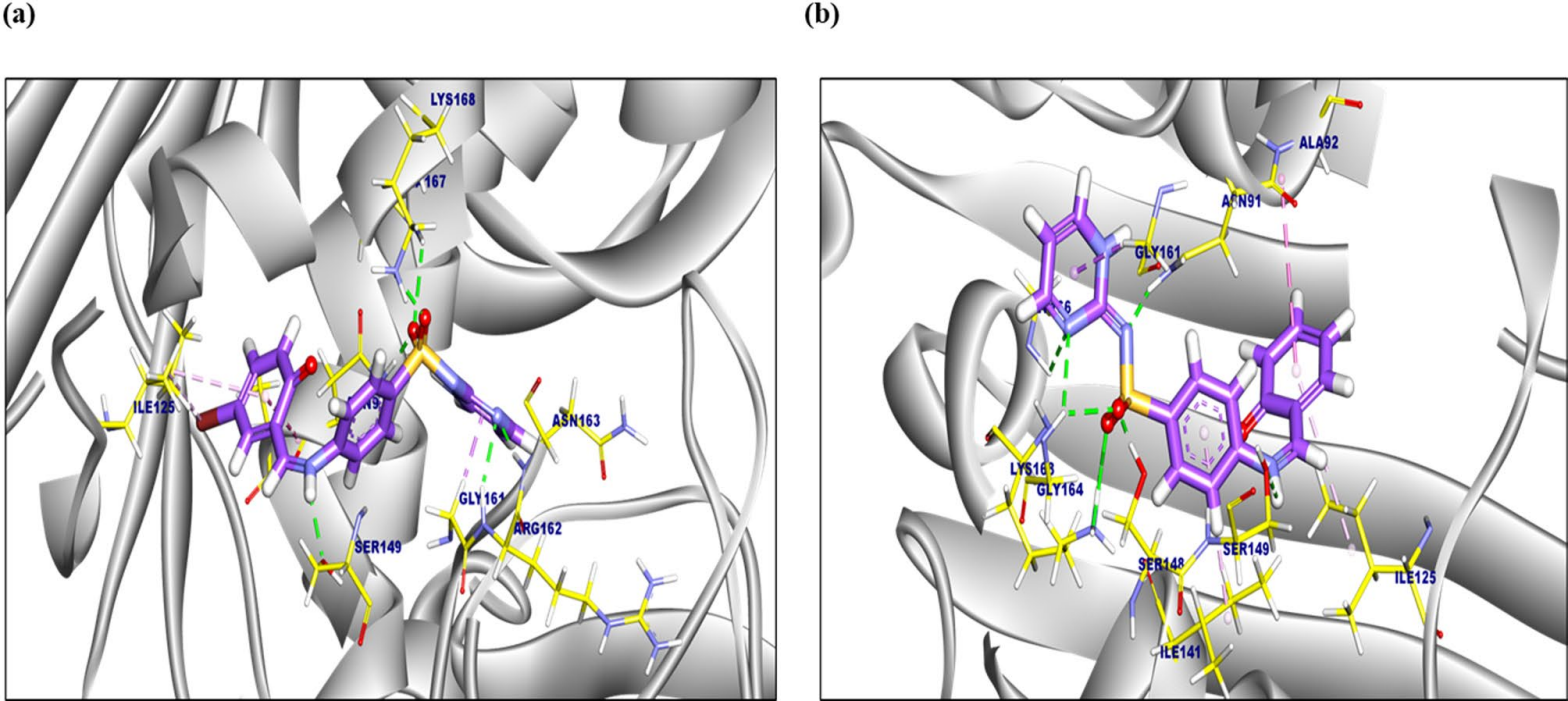
(**A**) 3D figure of the proposed binding mode of compound 1 against Human DNA topoisomerase II ATPase target site, amino acids colored by yellow, and the ligand colored by purple. (**B**) 3D figure of the proposed binding mode of compound 2 against Human DNA topoisomerase II ATPase target site, amino acids colored yellow, and ligand-colored purple.

The binding mode of SB1(Table 3) displayed a binding affinity of − 8.12 kcal/ mol. against *Human DNA topoisomerase II ATPase target site*. SB1 formed four hydrophobic π-alkyl, π-amide, and π-sigma interactions with Asn91, Ile125, and Gly161. Moreover, five hydrogen bonds were observed with Ala167, Asn91, Ser149, Lys168, and Arg162 with distances of 2.59, 1.90, 2.07, 2.04, 1.99, and 2.22 Å (Fig. 13a). Moreover, the proposed binding mode of SB2 (Table 3) exhibited an affinity score of -8.56 kcal/mol. Four π-alkyl, π-amide, and π-sigma interactions were conducted with Gly161, Asn91, and Ile141. Additionally, it formed seven hydrogen bonds with Asn91, Lys168, Ser148, Gly164, Gly166, and Ser149 with distances of 2.28, 2.66, 1.83, 2.38, 2.91, 2.82 and 2.91 Å (Fig. 13b).

**Table 2:** Molecular docking analysis of the tested compounds against carbonic anhydrase XII.

Target proteins	Tested compounds	RMSD value (Å)	Docking (Affinity) score (kcal/mol)
Carbonic anhydrase XII	SB1	1.57	− 6.12
	SB2	1.23	− 7.95
	Co-crystalized ligand	0.81	− 6.76

**Table 3:** Results of Molecular Docking Analysis of the tested Compounds against Human DNA topoisomerase II ATPase target site.

Target proteins	Tested compounds		
		RMSD value (Å)	(Affinity) score (kcal/mol)
Human DNA topoisomerase II ATPase target site	SB1	1.35	− 8.12
	SB2	1.12	− 8.56

## Discussion

Cancer is a multi-stage disease that is a major global health concern. It results from abnormalities that interfere with regular cellular functions, causing uncontrolled cell division and apoptosis evasion. The need for novel, tailored anticancer drugs is highlighted by the side effects of chemotherapy and other modern treatments, despite their effectiveness. The diverse biological activities of Schiff bases, a class of heterocyclic compounds, have attracted attention. These activities include antibacterial, antioxidant, and anticancer properties. Their imine group and functional architecture enhance their potential for therapeutic application. The current research aims to synthesize Schiff base compounds and evaluate their anticancer effects on various human cancer cell lines in order to develop more effective cancer treatments.

The results obtained showed that SB1 and SB2 have moderate anticancer effects against various human cancer cell lines, including HCT116, MCF-7, A549, HepG2, T24. These effects were confirmed by employing cytotoxicity assays, apoptosis induction, oxidative stress measurements, DNA fragmentation, and inhibition of TOPO I and II. SB2 consistently exceeded SB1 in terms of cytotoxicity, ROS generation, DNA fragmentation, and topoisomerases enzyme inhibition. This superior action, which leads to enhanced cellular absorption, greater binding affinities, or increased intracellular stability, is believed to be caused by structural differences between the two compounds. A significant factor influencing the SB2 anticancer properties is the presence of bromine atom in -N-(pyrimidin-2-yl) benzene sulfonamide. Bromine has a variety of effects on cancer cells due to its electrical and steric properties. It has been noted that bromine alters the electrical properties of the compounds, enhancing their capacity to engage with biological targets and boosting their anticancer efficacy. Adding a bromine atom to Schiff bases boosts their cytotoxic effects on cancer cells while also increasing their lipophilicity and cellular absorption. This suggestion comes in agreement with previous reports showing the anticancer activity is substituent dependent, brominated derivatives^21,41^

Flow cytometry analysis showed that SB1 and SB2 significantly increased early apoptotic cells populations (Figs. 2 and 3). Following treatment with SB1 and SB2, early apoptosis reached 11.72%, 17.13% in MCF-7 cells, and 14.86%, 19.18% in HCT116 cells respectively, compared to controls MCF-7 (0.37%) and HCT116 (0.66%). The results show efficient induction of programmed cell death, which may be related to the activation of intrinsic apoptotic pathways, possibly caused by mitochondrial malfunction, oxidative stress and DNA damage^42^. Interestingly, SB2 elicited a more potent apoptotic response than SB1 in both cell lines, consistent with its stronger cytotoxic action. Structural changes such as bromine substitution may contribute to this improved impact which may affect lipophilicity and cellular absorption.

Mechanistically, SB1 and SB2 modulated key regulators of the intrinsic apoptotic pathway. Elevated cytochrome c levels indicate mitochondrial outer membrane permeabilization, a critical step leading to apoptosome formation and caspase activation^43^. This is supported by an increased Bax/Bcl-2 ratio, reflecting a shift toward a pro-apoptotic state and enhanced mitochondrial destabilization^44^. Consistent with activation of the intrinsic pathway, significant activation of caspase-9 was detected in HCT116 and MCF-7 cells, followed by activation of the executioner caspases-7. Caspase-3 activation was observed only in HCT116 cells, aligning with the known deficiency of functional caspase-3 in MCF-7 cells, this observation aligns with previous reports that MCF-7 cells lack functional caspase-3 and instead rely on alternative executioner caspases such as caspase-7^45^.

Further evidence of apoptosis induction was shown by enhanced intracellular ROS formation (Fig. 5) and DNA fragmentation (Fig. 6), both of which are hallmarks of mitochondrial-mediated apoptosis. In HCT116 cells, treatment with SB1 and SB2 caused a significant increase in DNA fragmentation by about 7.3- and 10.0-fold respectively and in MCF-7 cells 6.3- and 8.9-fold compared to controls. Similar to earlier findings, SB2 caused more DNA fragmentation in both cell lines than SB1, suggesting a more robust DNA-damaging action.

Consistent with these findings, intracellular ROS levels were significantly elevated following treatment. In MCF-7 cells, ROS levels increased by approximately 31.7% and 46.2% after treatment with SB1 and SB2, respectively, while in HCT116 cells, ROS levels increased by 27.1% and 35.3%, respectively, relative to untreated controls. The more pronounced ROS generation observed with SB2 suggests enhanced induction of oxidative stress, which is known to promote DNA damage and amplify apoptotic signaling pathways. The DPA assay further confirmed the ability of both compounds to induce DNA fragmentation, reinforcing their role in apoptosis induction^46^. These results are consistent with MTT assay findings and support DNA damage-mediated apoptosis as a key contributor to the greater cytotoxicity observed for SB2.

The significant reduction in topoisomerases enzyme activity showed that SB1 and SB2 inhibited TOPO I and II. SB2 had more inhibitory effects than SB1, reducing TOPO I and II levels in a dose-dependent fashion. Cell death and the accumulation of DNA damage might result from the blocking of these enzymes, which are necessary for transcription and DNA replication. The synthesized compounds may inhibit topoisomerases by binding to the enzyme–DNA complex and preventing DNA strand religation. The presence of bromine in SB2 enhances the compound’s ability to bind to the enzyme and enhances its inhibitory effects^47,48^.

The potential anticancer relevance of these compounds was further supported by target prediction, which indicated that they might operate through a multi-target mechanism including cancer-related proteins like matrix metalloproteinases, BRAF, CDK2/Cyclin A, and carbonic anhydrase isoforms. However, ADMET analysis revealed pharmacokinetic limitations, including low solubility, limited membrane permeability, high plasma protein binding, and short half-life, which may affect bioavailability. Therefore, even SB1 and SB2 show promising in silico anticancer potential, further structural optimization and experimental validation are necessary to improve their drug-like properties and confirm their therapeutic efficacy^49^.

Molecular docking study showed that SB1 and SB2 had favorable binding affinities for human DNA topoisomerase II ATPase and Carbonic anhydrase XII, with interaction patterns that were in line with previously reported Schiff base inhibitors. SB1 showed strong affinity toward CA XII, including possible zinc coordination, while SB2 exhibited slightly better stabilization within the topoisomerase II ATP-binding pocket^50,51^.

## Conclusion

Schiff bases have moderate in vitro antineoplastic action and have good binding affinities for key proteins involved in cancer progression. The presence of a bromine atom in SB2’s chemical structure may be the reason for its higher effectiveness. Given its wide atomic radius and high electronegativity, the bromine atom probably improves SB2’s lipophilicity and electron-withdrawing capacity. Mechanistically, SB1 and SB2 induce apoptosis through activation of the intrinsic mitochondrial system, defined by alteration of Bax/Bcl-2 balance, release of cytochrome c, activation of caspase cascade, increased ROS production and DNA fragmentation. Notably, SB2 consistently yielded larger effects across these parameters, indicating a more robust pro-apoptotic profile. These observations were further substantiated by molecular docking investigations, which demonstrated favorable binding interactions of SB2 with tumor-associated proteins such as carbonic anhydrase XII and DNA topoisomerase II, possibly underlying its increased biological activity. Significantly, SB2 exhibited cancer cell specific cytotoxicity with minimal effects on normal cells, which underscores its therapeutic significance. Collectively, these findings show that SB2 has a well-defined mechanism of action, making it an attractive lead molecule for further preclinical anticancer research.

## Supplementary Information

Below is the link to the electronic supplementary material.

Supplementary Material 1

## Data Availability

The datasets used and/or analysed during the current study available from the corresponding author on reasonable request.
