# Supplementary material for: Synthesis, characterization, and anticancer potency of sulfadiazine salicylaldehyde-based Schiff bases
Source: Sci Rep. 2026 May 13;16:15047. doi: 10.1038/s41598-026-51752-z (PMC13172555; doi:10.1038/s41598-026-51752-z)
Supplement: Supplementary file 1 — Supplementary Material 1 [file 41598_2026_51752_MOESM1_ESM.docx]

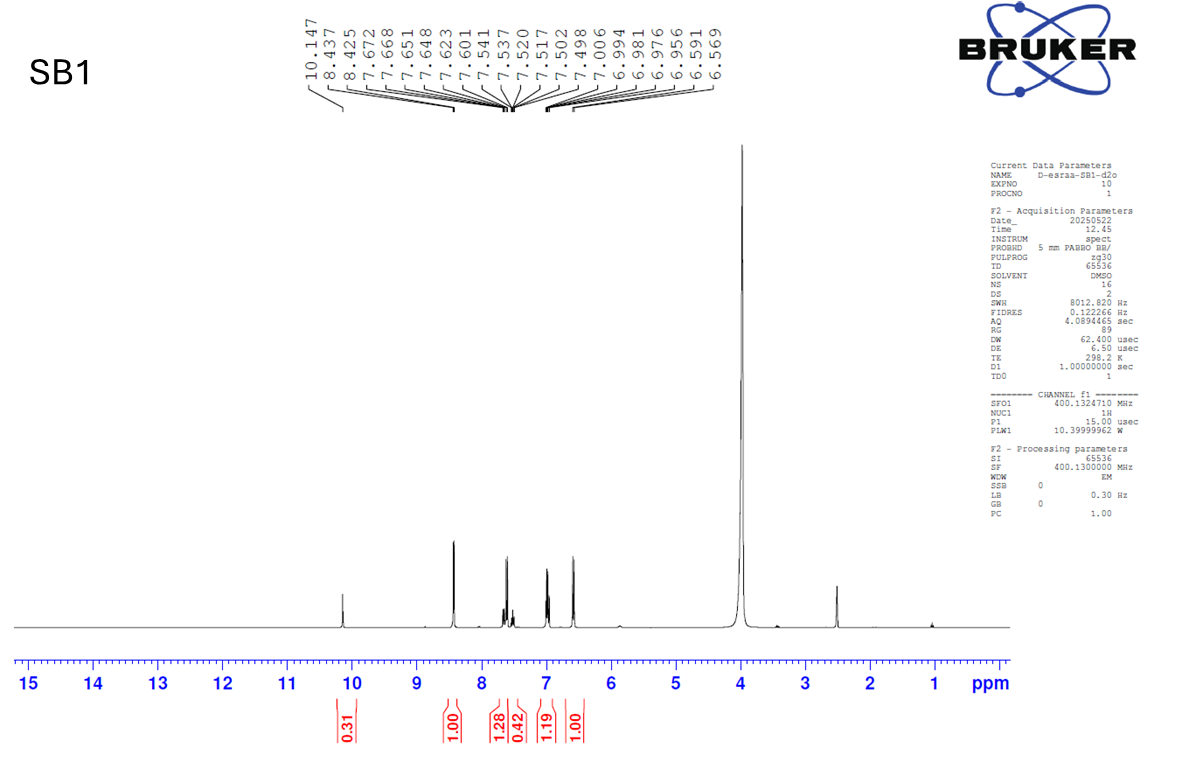


**Figure 1:** ^1^H NMR spectra analysis of SB1

**Figure 2:** ^13^C NMR spectra analysis of SB1


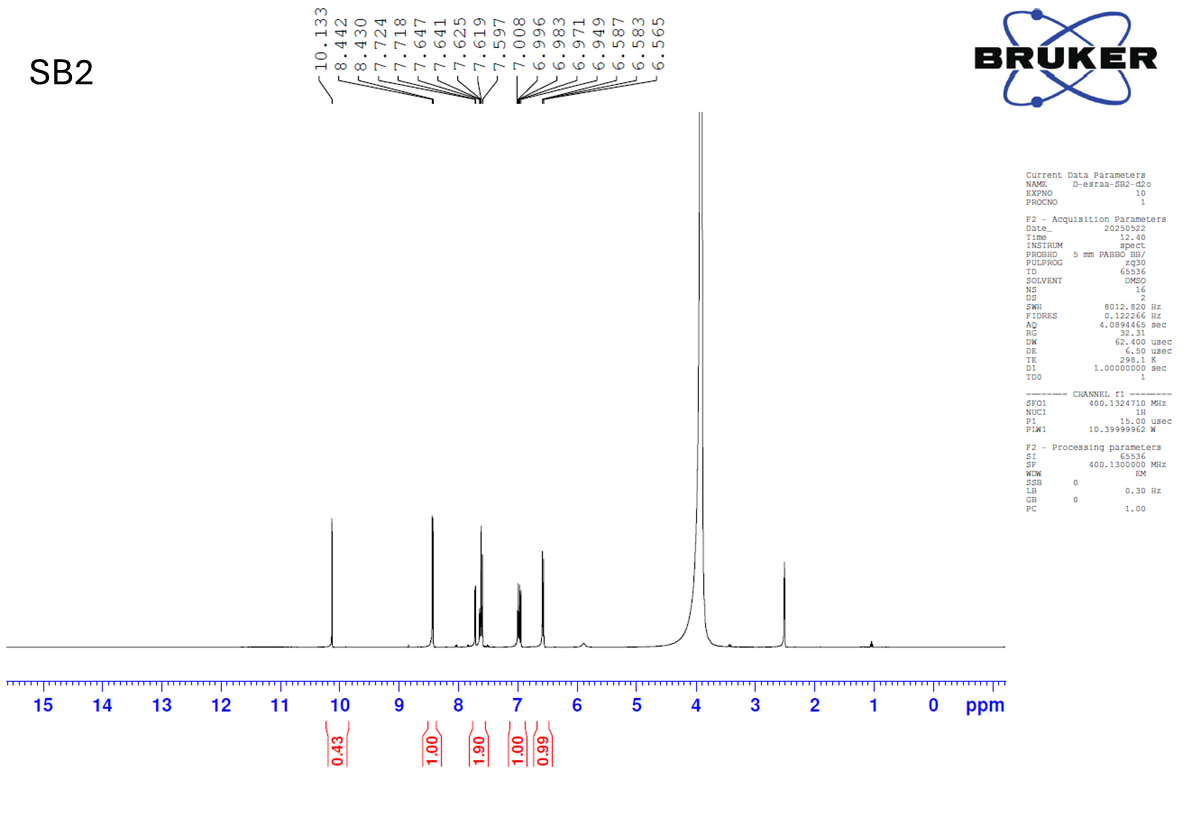


**Figure 3:** ^1^H NMR spectra analysis of SB2

**Figure 4**: ^13^C NMR spectra analysis of SB2
